# Effects of *Helicobacter pylori* infection on intestinal microbiota, immunity and colorectal cancer risk

**DOI:** 10.3389/fcimb.2024.1339750

**Published:** 2024-01-26

**Authors:** Veronika Engelsberger, Markus Gerhard, Raquel Mejías-Luque

**Affiliations:** Institute for Medical Microbiology, Immunology and Hygiene, TUM School of Medicine and Health, Department Preclinical Medicine, Technical University of Munich, Munich, Germany

**Keywords:** *Helicobacter pylori*, colorectal cancer, intestinal microbiome, immune response, eradication therapy, antibiotics

## Abstract

Infecting about half of the world´s population, *Helicobacter pylori* is one of the most prevalent bacterial infections worldwide and the strongest known risk factor for gastric cancer. Although *H. pylori* colonizes exclusively the gastric epithelium, the infection has also been associated with various extragastric diseases, including colorectal cancer (CRC). Epidemiological studies reported an almost two-fold increased risk for infected individuals to develop CRC, but only recently, direct causal and functional links between the chronic infection and CRC have been revealed. Besides modulating the host intestinal immune response, *H. pylori* is thought to increase CRC risk by inducing gut microbiota alterations. It is known that *H. pylori* infection not only impacts the gastric microbiota at the site of infection but also leads to changes in bacterial colonization in the distal large intestine. Considering that the gut microbiome plays a driving role in CRC, *H. pylori* infection emerges as a key factor responsible for promoting changes in microbiome signatures that could contribute to tumor development. Within this review, we want to focus on the interplay between *H. pylori* infection, changes in the intestinal microbiota, and intestinal immunity. In addition, the effects of *H. pylori* antibiotic eradication therapy will be discussed.

## 1 Introduction

### 1.1 Helicobacter pylori

*Helicobacter pylori* is a gram-negative, microaerophilic, spiral-shaped bacterium that colonizes the gastric mucosa of 43% of the world’s population (Malfertheiner et al., 2023). Infection rates depend on the geographical location, with high variation among regions reflecting urbanization, sanitation, access to clean water, and socioeconomic status (Hooi et al., 2017). It is suggested that *H. pylori* infection is acquired during early childhood through oral-oral or fecal-oral transmission routes and usually persists lifelong. Spontaneous clearance without encountering antibiotics is seldom (Yokota et al., 2015; Kayali et al., 2018). *H. pylori* was first cultivated from the stomach and characterized in 1984 by Barry Marshall and Robin Warren, who linked the bacterium to chronic gastritis and gastric and duodenal ulcers (Marshall and Warren, 1984). For this discovery, which reversed the assumption that the stomach is a sterile organ because of its high acidity, Marshall and Warren were awarded the Nobel Prize in 2005 (Pincock, 2005). The milestone discovery also led to the cure of gastric pathologies by *H. pylori* eradication treatment, whose efficacy was first demonstrated in 1990 (Rauws and Tytgat, 1990). Further studies confirmed *H. pylori* as the underlying cause of gastritis (Morris and Nicholson, 1987; Graham et al., 1988). Notably, *H. pylori* infection was also identified as the strongest known risk factor for gastric cancer, as up to 90% of gastric cancer cases could be attributable to the infection. Chronic gastritis can result in gastric carcinogenesis, most often through the Correa cascade of atrophic gastritis, intestinal metaplasia, and dysplasia (Moss, 2017). In addition, the infection can lead to the development of gastric mucosa-associated lymphoid tissue (MALT) lymphoma (Pereira and Medeiros, 2014). For this reason, the International Agency for Research on Cancer classified *H. pylori* in 1994 as a class I carcinogen for gastric cancer and MALT lymphoma (Møller et al., 1995). Although 80% of infected individuals remain asymptomatic and are unaware of the presence of *H. pylori* in their stomachs, the infection always leads to chronic gastric inflammation. For some individuals, the infection can have severe consequences, leading to high morbidity and mortality (Suerbaum and Michetti, 2002). As a consequence, it is recommended that all *H. pylori*-infected patients should be treated with eradication therapy (Malfertheiner et al., 2022). Still, the susceptibility to developing severe illness depends on *H. pylori* strains and their virulence factors, environmental factors, and host genetics, such as inadequate T-cell responses (Robinson et al., 2008).

**Figure 1:**
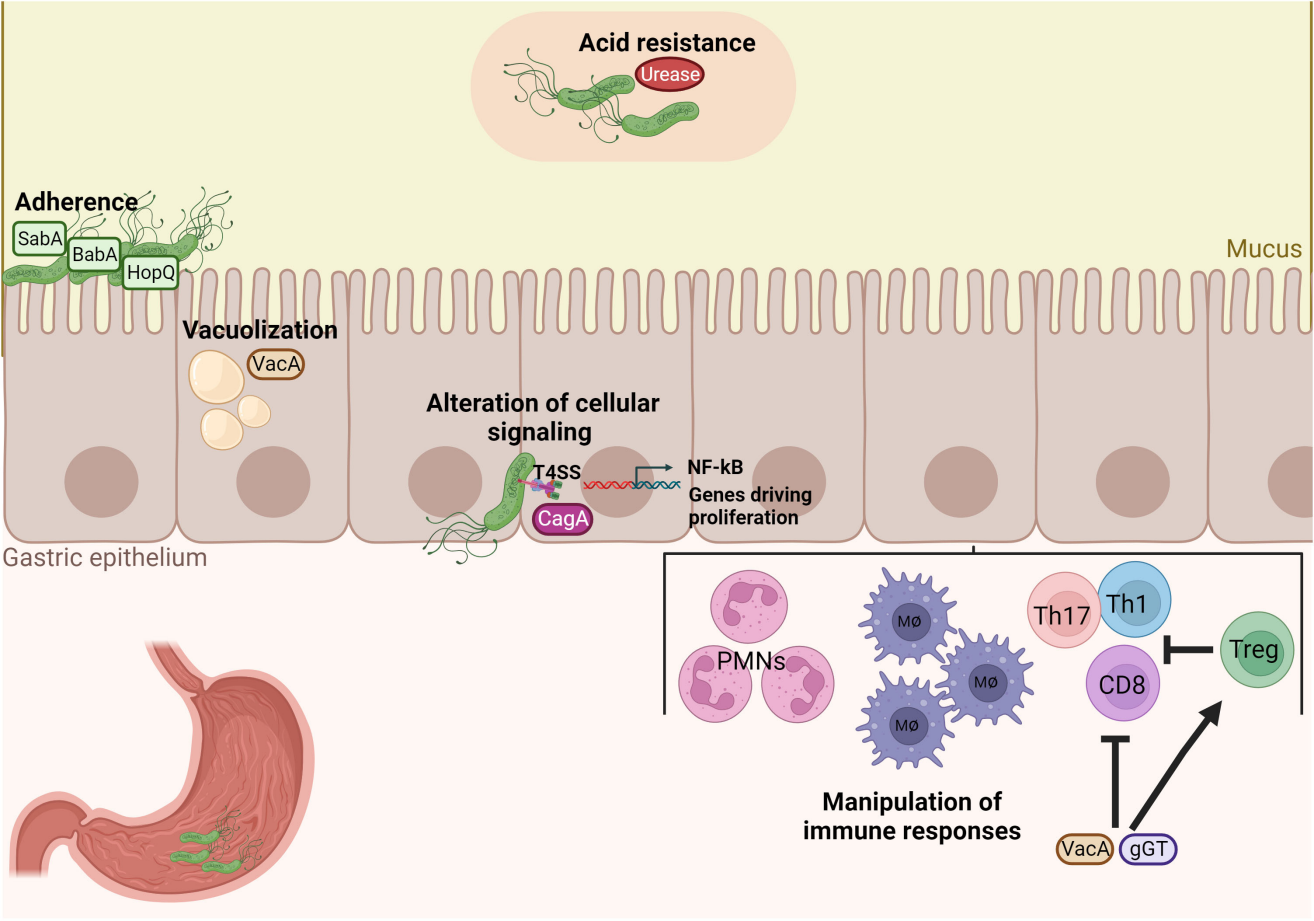
*H. pylori* possesses unique properties and virulence factors for successful colonization and manipulation of the host’s immune system. Acid neutralization by the enzyme urease and adherence factors of *H. pylori*, such as BabA, SabA and HopQ, are important for colonization of the gastric mucosa. Damage of gastric epithelial cells by vacuolization occurs by the virulence factor VacA and inflammation and pro-carcinogenic signaling is then induced by the cytotoxin-associated antigen pathogenicity island (CagPAI). Finally, VacA and gGT manipulate immune responses by inhibiting both CD4^+^ and CD8^+^ T cell responses and promoting the expansion of Treg cells in order to enable bacterial persistence.

*H. pylori* has evolved several mechanisms, mainly exerted by specific virulence factors, for successful colonization of the human stomach, manipulation of the host immune system, and signaling pathways by simultaneously avoiding clearance and maintaining a chronic inflammatory infection (**Figure 1**). First, after entering the unique hostile environment of the stomach, *H. pylori* uses its helical shape and motility driven by a bundle of flagella to reach the viscous gastric mucosa and for initial colonization (Ottemann and Lowenthal, 2002) (Martínez et al., 2016). Secondly, *H. pylori* possesses the enzyme urease to neutralize the acidic gastric environment (pH 2) by converting urea to ammonium and carbonic acid (Weeks et al., 2000). After reaching the gastric mucus layer, *H. pylori* adheres to gastric epithelial cells using adhesins such as the blood group antigen binding adhesin (BabA) and the sialic acid binding adhesin (SabA). Those adhesins mediate binding interactions with fucosylated Lewis b histo-blood group antigens and sialyl-Lewis x antigens, respectively (Mahdavi et al., 2002; Rad et al., 2002). Upon chronic inflammation, carcinoembryonic antigen-related cell adhesion molecules (CEACAM) receptors are expressed in the stomach mucosa and support further adhesion by interacting with *Helicobacter* outer membrane protein (Hop) Q. The HopQ-CEACAM interaction also enables the translocation of the virulence factor cytotoxin-associated gene A (CagA) into host cells (Javaheri et al., 2016; Königer et al., 2016). CagA is one of the most critical factors of *H. pylori*. CagA is injected into host gastric epithelial cells by a type IV secretion system (T4SS) encoded by the 40-kb cag pathogenicity island (CagPAI) (Xiang et al., 1995; Censini et al., 1996; Odenbreit et al., 2000). Upon translocation, differential phosphorylation of CagA by c-Src and c-Abl kinases activates its effector properties, including perturbation of multiple cell signaling pathways, thereby promoting tumorigenesis of gastric cancer (Mueller et al., 2012; Yong et al., 2015). Numerous studies showed that, in particular, CagA-positive *H. pylori* strains induce pro-inflammatory and pro-carcinogenic effects on gastric epithelial cells, resulting in peptic ulcer, premalignant lesions, and gastric adenocarcinoma (Blaser et al., 1995; Parsonnet et al., 1997; Huang et al., 2003). Thus, CagA is also designated as an oncoprotein of *H. pylori*. Other important virulence factors secreted by *H. pylori* are the pore-forming Vacuolating cytotoxin A (VacA) and the γ-glutamyl transpeptidase (γGT). VacA can induce cell apoptosis and subsequently inhibit T-cell activation (Boncristiano et al., 2003; Palframan et al., 2012). Lastly, γGT is an enzyme found in nearly all *H. pylori* strains and participates in glutamate metabolism. γGT damages gastric epithelial cells and further contributes to immune tolerance (Schmees et al., 2007; Shibayama et al., 2007; Käbisch et al., 2016). Altogether, those virulence factors are critical for the colonization and persistence of *H. pylori* in the human host.

### 1.2 *H. pylori* and extragastric manifestations

Although *H. pylori* chronically persists in a unique niche, the human stomach, where it it maintains an intrincate host-pathogen relationship, the infection has also been associated with systemic effects and a variety of extragastric diseases in the past years (Banić et al., 2012). Mainly, epidemiologic studies demonstrated that *H. pylori* infection negatively affects several diseases, such as neurological and coronary heart disorders, but also exerts protective effects on the host, for example, by reducing the risk for asthma and inflammatory bowel disease (IBD) (Kyburz and Müller, 2017).

Besides being the main risk factor for gastric cancer, the infection was associated with an increased risk for other digestive cancers, such as colon cancer, pancreatic cancer, and hepatocellular carcinoma (Xiao et al., 2013; Butt and Epplein, 2019; Madala et al., 2021). Outside of the gastrointestinal (GI) tract, *H. pylori* is reported to affect neurological, hematologic, and metabolic diseases. The association is less clear for dermatological, ocular, cardiovascular, and allergic diseases (Franceschi et al., 2014; Gravina et al., 2018; He et al., 2022). In contrast, *H. pylori* infection was also shown to protect from chronic inflammatory diseases, such as IBD. One mechanism described in IBD protection involves the manipulation of dendritic cells (DC) by *H. pylori*. DCs are major antigen-presenting cells and essential for activating naïve T lymphocytes (Th0) to differentiate into different T-cell subsets (Hilligan and Ronchese, 2020). It was shown that *H. pylori* impairs the maturation of DCs and induces a tolerogenic phenotype resulting in high numbers of regulatory T-cells (Treg cells). Mechanistically, CagA activates the signal transducer and activator of transcription 3 (STAT3) signaling in human DCs, inducing tolerization of DCs and subsequently favoring a tolerogenic immune response in the infected host (Kaebisch et al., 2014). Besides CagA, γGT was also demonstrated to promote a regulatory immune response in *H. pylori*-infected gastric tissue by provoking a semimature state of DCs, which was dependent on glutamate produced as result of γGT activity (Oertli et al., 2013; Käbisch et al., 2016). It is believed that *H. pylori*-induced Treg cells secreting anti-inflammatory interleukin (IL)-10 and transforming growth factor ß (TFGß) can migrate to other sites of the human body and exert immunoregulatory effects, thereby reducing the risk for inflammatory conditions and IBD. In addition, *H. pylori* stimulates a systemic immunosuppressive T helper 2 (Th2)/macrophage M2 response and activates the NLR family pyrin domain containing 3 (NLRP3) inflammasome, followed by IL-1ß and IL-18 cytokine secretion, which, together with a Treg-skewed response, protects from the development of IBD (Engler et al., 2015; Feilstrecker Balani et al., 2023). The protective effect on IBD with convincing experimental evidence suggests a pivotal role of *H. pylori* infection on systemic immune responses.

However, the association between *H. pylori* infection and extragastric diseases is mainly based on epidemiological studies, and underlying mechanisms for most extragastric diseases are still unclear and currently under investigation. One possibility could be that the bacterium exerts direct effects, which would imply that the bacterium can colonize organs other than the stomach. Due to the unique adaption of *H. pylori* to the gastric mucosa, different habitats in the human body seem unlikely. Studies investigating other sites of *H. pylori* colonization than the stomach are sparse. Regarding the colon, only a few studies could detect *H. pylori* genetic sequences by qPCR or sequencing in human colon biopsies (Grahn et al., 2005; Monstein et al., 2005; Keenan et al., 2010), and this is most likely due to the transit of non-viable but not of resident bacteria. Kienesberger et al. could not detect *H. pylori* in murine feces by qPCR or by high-throughput sequencing of fecal, ileal, and cecal samples after six months of infection (Kienesberger et al., 2016). In humans, Vasapolli et al. analyzed the transcriptionally active microbiota of the whole GI tract in *H. pylori* positive and negative patients. However, they could not detect 16S rRNA of *H. pylori* in the lower GI tract or feces, suggesting that *H. pylori* is not an active member of the microbiota in the small and large intestine (Vasapolli et al., 2019). Therefore, it seems plausible that *H. pylori* infection affects other organs, particularly the colon, through systemic immunomodulation, and alteration of microbial composition and function (He et al., 2022). Those mechanisms will be discussed in more detail in the following sections.

Nevertheless, studies focusing on the effects of *H. pylori* in extragastric diseases present conflicting and heterogeneous results, which may be due to differences in age and ethnicity of the study population or due to different methods for *H. pylori* detection. For example, if *H. pylori* positivity is only defined by serological screening, *H. pylori*-eradicated patients might be counted as currently infected, which leads to a bias in the analyses.

## 2 *H. pylori* and colorectal cancer risk

### 2.1 Epidemiologic associations

The impact of *H. pylori* infection on increased CRC risk has been studied for years. Already in 1997, a small case-control study reported that in patients with colon polyps, *H. pylori* prevalence, measured by IgG antibody responses, was significantly higher than in matched controls (49% in controls vs. 71.4% in patients with colon polyps, OR=2.6). However, in patients with colon cancer, *H. pylori* incidences (69.2%, p=0.18) did not significantly differ from those of control patients, which might be due to antibiotic treatment of CRC patients before surgery and, thus, unintentional *H. pylori* eradication (Meucci et al., 1997). Later, a study from Korea reported that patients with colorectal neoplasms had a significantly higher *H. pylori* prevalence than control patients, resulting in an OR of 1.90 (Nam et al., 2013). When analyzing Chinese patients undergoing both colonoscopy and esophagus-gastro-duodenoscopy, it was found that *H. pylori* infection and *H. pylori*-associated gastric diseases doubled the risk for colorectal neoplasia, but the severity of gastric conditions was only mildly correlated with the level of CRC risk (Qing et al., 2016). Kim et al. could also confirm a positive association between *H. pylori* and CRC risk. In a large-scale study involving around 9000 healthy male subjects undergoing screening colonoscopy and testing for *H. pylori* IgG antibody response, the odds for advanced colorectal neoplasm in *H. pylori*-positive participants was 1.90 (Kim et al., 2017). In a Chinese cohort with almost 4000 *H. pylori*-positive individuals, Liu et al. reported a significantly higher risk of developing CRC (hazard ratio (HR)=1.87) as well, the risk of CRC was associated with the frequency of *H. pylori*-related clinical visits. Of note, *H. pylori*-infected participants also displayed a much higher number of other comorbidities, such as hypertension, diabetes mellitus, hypercholesterolemia, and coronary artery disease (Liu et al., 2019). In 2022, a meta-analysis of seventeen observational studies summarized that *H. pylori* infection is an independent risk factor for overall colorectal polyps with an OR of 1.67. In particular, the infection is associated with adenomatous polyps (OR=1.71), advanced adenomatous polyps (OR=2.06), and hyperplastic polyps (OR=1.54) but not with sessile serrated polyps (OR=1.00) (Lu et al., 2022). Another systematic review and meta-analysis came to similar results. Wu et al. found a positive association between *H. pylori* and the risk for colorectal adenoma (OR=1.66), as well as for CRC (OR=1.39) (Wu et al., 2013). A summary OR for the relationship between *H. pylori* infection and CRC risk of 1.4 was found by a smaller and earlier meta-analysis. The lower OR from this meta-analysis could be due to the very small and heterogeneous studies available in those early years (Zumkeller et al., 2006). Finally, the up-to-date greatest retrospective population-based study from the USA reported an independent positive association between the history of *H. pylori* infection status and CRC development with an OR of 1.89. However, as the *H. pylori* status was determined based on serological testing, the authors could not differentiate between active or past infection and thus explore the effects of eradication therapy on CRC risk (Boustany et al., 2023).

Certain virulence factors of *H. pylori* are associated with more severe gastric malignancies, predisposing some *H. pylori* carriers to a high risk for serious clinical outcomes. Whether this also accounts for the *H. pylori*-associated CRC risk is currently a subject of research. Butt et al. investigated, in a consortium of ten different US cohorts representing diverse populations, whether *H. pylori*-specific antigen responses were associated with CRC risk. The authors concluded that VacA seropositivity slightly increased the odds of CRC in a dose-response relationship (OR=1.11), which was especially strong for African Americans (OR=1.45) (Butt et al., 2019). Similarly, in a European study cohort, *H. pylori* positivity increased the risk for CRC with an overall OR of 1.36. Here again, antibody responses towards 13 *H. pylori* virulence factors were measured, and it appeared that the increased cancer risk was mainly driven by VacA (OR=1.34) and cysteine-rich protein C (HcpC) seropositivity (OR=1.66) (Butt et al., 2020). Other studies investigated the association between infection with *H. pylori* CagA-positive strains and CRC risk, as it is known that CagA-positive strains highly increase the risk for gastric cancer, as mentioned before. Indeed, Shmuely et al. found, in a relatively small Israelian cohort, CagA seropositivity to be strongly associated with an increased risk for not only gastric cancer (OR=88.1), as expected but also for CRC (OR=10.6) (Shmuely et al., 2001). Those findings were confirmed in another cohort with around 1000 CRC patients in China. CRC patients had higher *H. pylori* infection rates compared to control patients, and importantly, serum gastrin levels and expression of the proliferation marker Ki67 in colonic tumor tissues were significantly higher in patients infected with CagA-positive strains compared to CagA-negative strains (Zhang et al., 2020). These studies were the first to suggest that the effects of specific virulence factors, especially CagA and VacA, might not be limited to the stomach but influence inflammatory and carcinogenic processes in the gut. However, further studies are needed to confirm the molecular role of these virulence factors in CRC development to elucidate if those could be used as biomarkers. Overall, considering the large number of cohort studies and meta-analyses performed in the last decades, *H. pylori* infection can be regarded as a relevant risk factor for CRC by increasing the odds by almost a factor two. Nevertheless, those studies did not clarify causal and functional mechanisms.

### 2.2 Crosstalk between *H. pylori* infection and intestinal immune responses

*H. pylori* infection of the stomach elicits a pro-inflammatory response by recruiting multiple innate and adaptive immune cell populations into the gastric mucosa, which is critical for developing *H. pylori*-induced pathologies. At the same time, the virulence factors mentioned above allow the bacterium to evade innate immune mechanisms and manipulate antigen presentation and T-cell responses to maintain a chronic infection. Noteworthy, *H. pylori* infection also induces systemically inflammatory immune responses in the host (Jackson et al., 2009). The increasing evidence linking *H. pylori* infection to CRC development suggests a complex immune crosstalk between the upper and lower digestive tract. Hence, it can be assumed that the infection provokes a pro-inflammatory condition in the gut by manipulating CD4^+^ and CD8^+^ T-cell responses that may increase CRC risk.

#### 2.2.1 *H. pylori* infection controls CD4^+^ T-cell functions in the intestine

**Figure 2:**
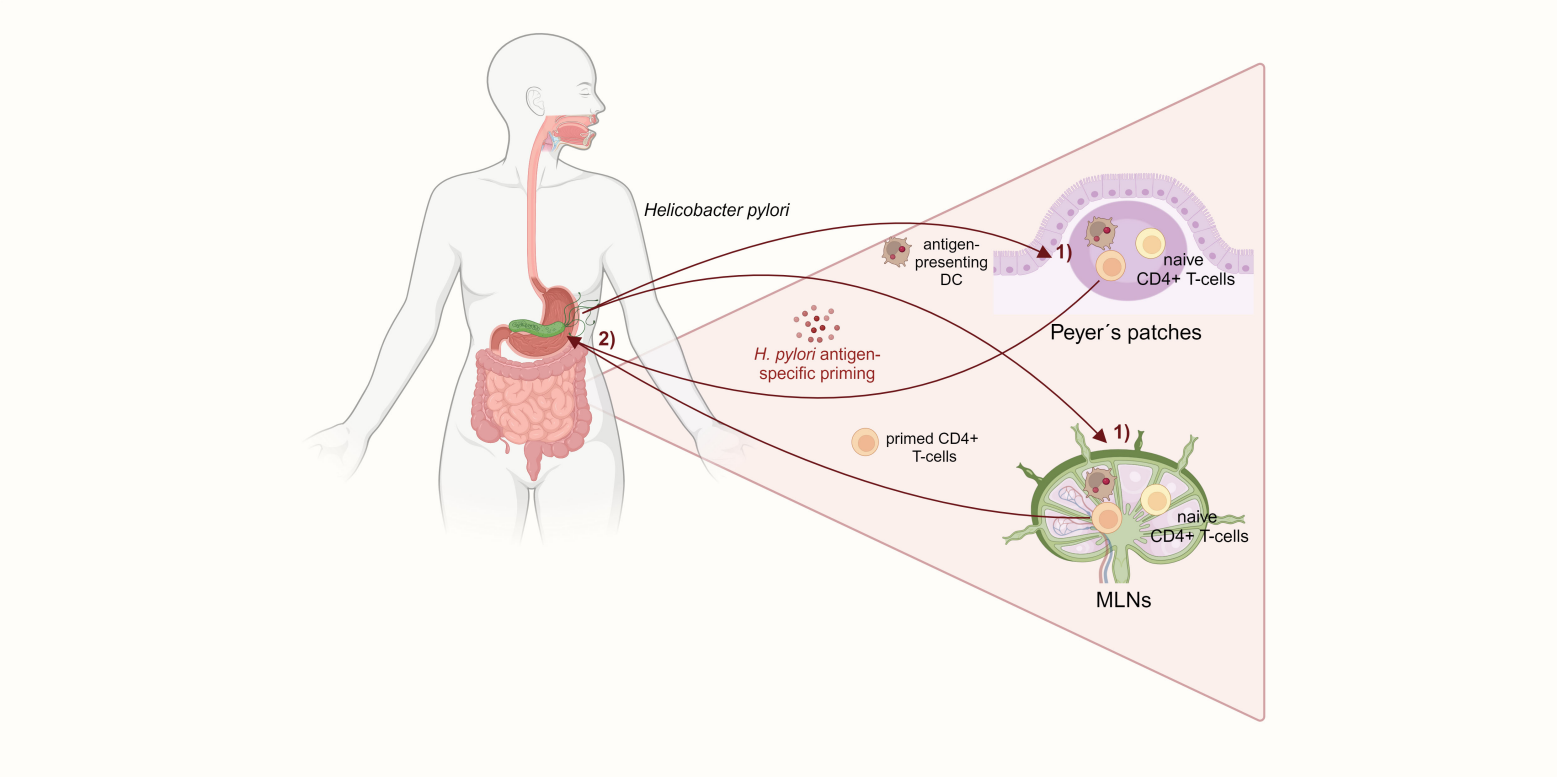
*H. pylori*-specific CD4^+^ T-cells are primed in the small intestinal Peyer´s patches and mesenteric lymph nodes. 1) DCs in the PPs and MLNs capture *H. pylori* and prime naïve CD4^+^ Tcells. 2) Those *H. pylori* antigen-specific CD4^+^ T-cells subsequently home to the gastric mucosa and induce gastritis. The activation of CD4^+^ T-cells in the small intestine, which is required for *H. pylori*-induced gastric inflammation, suggests a mechanism for extragastric immunomodulation.

It is well described that *H. pylori* induces a distinct CD4^+^ T-cell response in the gastric mucosa of the host. Initially, the infection promotes a mixed Th1 and Th17 response with a dramatic release of pro-inflammatory cytokines, such as interferon γ (IFNγ) and IL-17A (Hitzler et al., 2012). Upon uptake of *H. pylori* antigens, DCs increase CD83, CD86, and CD11c expression and promote a Th1 response with potent IFNγ secretion (Bimczok et al., 2010). In addition, IL-23-secreting DCs stimulate Th17 responses and IL-17 secretion against *H. pylori*, contributing to the infection pathology (Khamri et al., 2010). *H. pylori*-specific CD4^+^ T-cells, together with prolonged IFNγ secretion, were shown to be indispensable for gastritis, as mice lacking either *H. pylori*-specific CD4^+^ T-cells or IFNγ did not develop gastric inflammation. In addition, mice deficient in Treg cells suffered from a more severe gastritis (Gray et al., 2013). Treg cells allow life-long persistence of *H. pylori* by counteracting the initial pro-inflammatory Th1/Th17 response. Kao et al. showed that DCs located in the gastric lamina propria of mice highly expanded in numbers and got more activated after *H. pylori* infection. *H. pylori*-stimulated DCs induced a Treg-dominated immune response with downregulation of Th17 T-cells and IL-17 mRNA expression. The shifted Th17/Treg balance bypassed immune clearance of *H. pylori* (Kao et al., 2010). However, the question of how *H. pylori*-induced T-cell responses affect the small intestine and colon remains unclear. An essential step towards answering this question were findings by Nagai et al. in 2007. The authors showed in mice that Peyer’s patches (PPs) in the small intestine are indispensable for *H. pylori* antigen-specific priming of naïve CD4^+^ T-cells, which then home to the stomach and subsequently cause gastritis. PPs can phagocytose the coccoid form of *H. pylori*, whose antigens are then captured by DCs and prime CD4^+^ T-cells. These activated and *H. pylori*-specific T-cells migrate to the gastric mucosa and induce inflammation (Nagai et al., 2007). Of note, mesenteric lymph nodes (MLNs) have been determined as early priming sites of *H. pylori* Th1/Th17 responses, and it was speculated that *H. pylori*-specific T-cells might be primed in PPs and migrate to MLNs before homing to the gastric mucosa (Shi et al., 2010). Another study confirmed that mice lacking PPs do not develop Th1-mediated gastritis or produce antibodies after infection with *Helicobacter felis*, which provokes a similar immune response to *H. pylori*. By transferring splenic T-cells from C57BL/6 mice infected with *H. felis* into immunodeficient RAG2 knockout mice, it was found that infiltration of *Helicobacter*-specific CD4^+^ T-cells into the gastric mucosa depends on *Helicobacter* colonization in the stomach (Kiriya et al., 2007). PPs and MLNs are major induction sites of mucosal immunity in the gut, maintaining immune tolerance and exerting pathogen defense. Commensal gut microbiota provide stimuli required for homeostasis of conventional and regulatory CD4^+^ T-cells in MLNs and PPs, and breaking of this tolerance can cause chronic mucosal inflammation that precedes colon carcinogenesis, as seen in IBD (Maloy and Powrie, 2011; Cording et al., 2013). Therefore, it can be hypothesized that the consequences of *H. pylori* infection on intestinal immunity and inflammation might be induced by aberrant antigen sensing and T-cell induction in the PPs and MLNs (**Figure 2**). Indeed, alterations in CD4^+^ T-cell differentiation were recently found in the intestine of mice with mutations in the adenomatous polyposis coli (*Apc*) gene (*Apc* ^+/Min^ and *Apc* ^+/1638N^), which, upon *H. pylori* infection, developed twice as many tumors as non-infected controls (Ralser et al., 2023), reflecting the two-fold increased risk for CRC observed in epidemiologic human studies. Higher tumor burden resulted in reduced survival of infected *Apc*-mutant mice, which showed increased infiltration of pro-inflammatory CD3^+^ T-cells and reduced Treg cells in the small intestinal and colonic epithelium. This imbalance between pro-inflammatory and Treg cells may be responsible for the increased tumorigenesis observed in *H. pylori*-infected *Apc-*mutant mice. Moreover, *H. pylori* infection activated pro-inflammatory and pro-carcinogenic STAT3 signaling in the intestinal and colonic epithelium. Activation of STAT3 signaling is a major driver of *H. pylori*-induced gastric carcinogenesis and is also seen in CRC initiation and development (Corvinus et al., 2005; Balic et al., 2020). RNA transcriptomic analysis of the small intestine and colon using single-cell RNA-sequencing also revealed a re-programming of Treg cells towards a Th17 phenotype defined by significantly increased Th17 differentiation genes in activated Treg cells from *H. pylori*-infected mice. Therefore, it was shown that a sustained pro-inflammatory immune response with STAT3 signaling in the small intestine and colon, as well as the presence of pathogenic FoxP3^+^/IL-17^+^ T-cells, contribute to colon carcinogenesis driven by *H. pylori* infection (Ralser et al., 2023).

**Figure 3:**
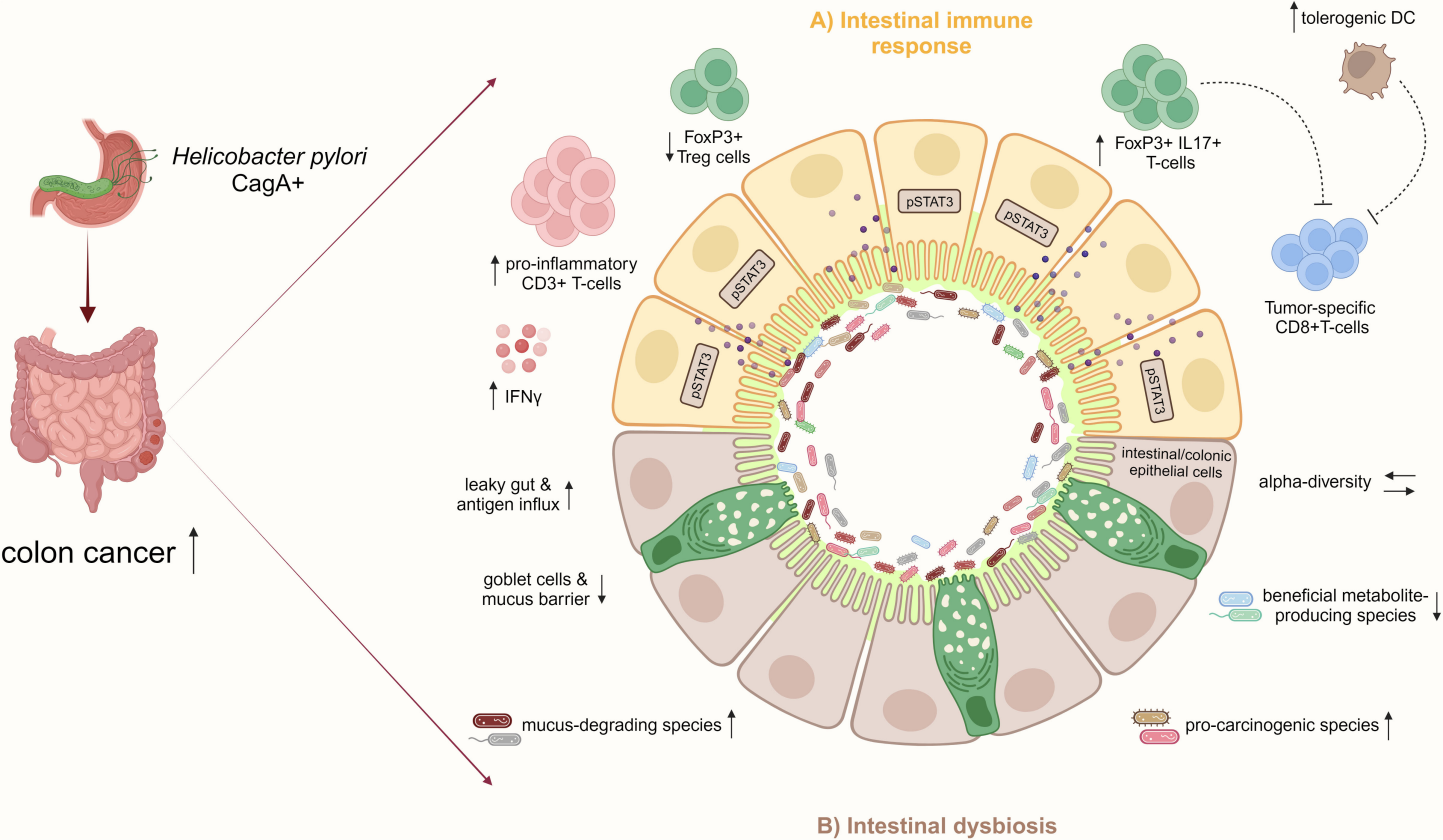
*H. pylori* infection increases the risk for CRC by inducing pro-inflammatory immune responses and intestinal microbiome changes. **(A)** In the small and large intestinal epithelia, represented schematically in the illustration, *H. pylori* infection, particularly with CagA positive strains, promotes a pro-inflammatory immune response characterized by CD3^+^ T-cell infiltration and IFNγ secretion and activates pro-carcinogenic pSTAT3 signaling. Concomitant with the reduction of Treg cells, the infection induces pathogenic FoxP3^+^/IL-17^+^ T-cells, which may dampen tumor-specific CD8^+^ T-cells. Also, the increase of regulatory DCs by *H. pylori* may impair CD8^+^ T-cell effector functions contributing to colon cancer. **(B)** Furthermore, *H. pylori* infection indirectly affects the intestinal microbiota by increasing mucus-degrading and pro-carcinogenic species and reducing beneficial metabolite-producing bacteria. Together with the loss of mucus-producing goblet cells and intestinal barrier impairment, the infection results in a leaky gut and increased antigen influx, which further manifests as inflammation in the epithelium. These *H. pylori*-induced disturbances of the intestinal immunity and microbiome promote CRC development.

The intestinal mucosal barrier is critical for maintaining homeostasis, and gut barrier disruption predisposes to colitis and CRC (Michael, 2019). It can be hypothesized that intestinal inflammation induced by *H. pylori* increases gut barrier leakage, facilitating an influx of antigens and immune-stimulating molecules and creating a pro-inflammatory vicious cycle. In a chronic DSS-induced colitis mouse model, infection with an *H. pylori* CagA positive strain aggravated disease activity and reduced intestinal mucosal integrity. CagA-containing exosomes mediated IFNγ-induced intestinal epithelial impairment and upregulated Claudin-2 expression, mediating a leaky gut (Guo et al., 2022). In summary, this study further relates CD4^+^ T-cell responses characterized by high levels of IFNy secretion to *H. pylori*-driven intestinal carcinogenesis (**Figure 3**), and it highlights CagA as the predominant virulence factor in *H. pylori*-associated CRC, as previously indicated by epidemiological studies.

#### 2.2.2 *H. pylori* infection controls systemic CD8^+^ T-cell functions

Although CD4^+^ T-cell responses toward *H. pylori* infection are well described, the role of CD8^+^ T-cells has so far mainly been neglected (Kronsteiner et al., 2014). However, CD8^+^ T-cells were identified as an essential source of IFNγ in *H. pylori*-infected individuals in an antigen-specific manner (Quiding-Järbrink et al., 2001). Only recently, Koch et al. showed that CD8^+^ T-cells with a tissue-resident phenotype specific for CagA control the bacterium through antigen-specific effector functions in the early infection phase. Later, during the chronic infection phase, CD8^+^ T-cells are replaced by CD4^+^ T-cells (Koch et al., 2023). Another study found that in mice deficient in CD4^+^ T-cells, the gastric mucosa was heavily infiltrated by CD8^+^ T-cells and leading to a more severe disease (Tan et al., 2008). This suggests that CD8^+^ T cells during *H. pylori* infection might be controlled by regulatory effector properties of CD4^+^ T cells. Moreover, CD4^+^ T cell-deficient mice did not have higher *H. pylori* colonization levels, implicating that CD8^+^ T cells are important players in controlling *H. pylori* growth (Tan et al., 2008).

Colon tumors with high infiltration of cytotoxic CD8^+^ T-cells have a better prognosis than low infiltrated tumors due to the critical role of cytotoxic CD8^+^ T cells in tumor control as well as in response to immunotherapy (Mlecnik et al., 2010; Fridman et al., 2012). A direct effect of *H. pylori* on CD8^+^ T cells during *H. pylori*-driven carcinogenesis has not been described so far. However, the observed *H. pylori*-dependent increase of FoxP3^+^/IL-17^+^ T-cells in the small intestine and colon of infected mice (Ralser et al.) could be a potential mechanism suppressing tumor-specific CD8^+^ T-cells in CRC as described before (Ma and Dong, 2011). In a recent study, Velin et al. reported the consequences of *H. pylori* infection on cancer immunotherapies by skewing systemic CD8^+^ T-cell responses (Oster et al., 2022). Using an MC38 colon adenocarcinoma model, the authors found *H. pylori* to increase tumor volumes of infected mice and also to reduce the efficacy of anticytotoxic T-lymphocyte-associated protein 4 (CTLA4) and/or programmed death ligand 1 (PDL-1), as well as anti-cancer vaccine treatment. These findings were confirmed in the azoxymethane (AOM) and DSS mouse model, in which administration of anti-CTLA4 antibody was less effective in reducing tumor numbers in the colon of *H. pylori*-infected compared to non-infected mice. Mechanistically, a decreased number and activation status of tumor-specific CD8^+^ T-cells in infected mice were observed. *In vitro* assays showed that DCs from infected mice promoted lower tumor-specific CD8^+^ T-cell proliferation. Surprisingly, those effects were independent of *H. pylori*-induced fecal microbiota changes and could not be rescued by antibiotic therapy. Together, it was shown that *H. pylori* systemically dampens tumor-specific CD8^+^ T-cell responses and impairs cancer immunotherapy. The skewed CD8^+^ T-cell response with reduced cytotoxic activity based on *H. pylori*-induced impairment of DC function may also have implications for colon carcinogenesis (**Figure 2**). Further studies must elucidate if dampened CD8^+^ T-cell effector functions following *H. pylori* infection, along with gut microbiota changes, contribute to CRC development.

### 2.3 *H. pylori* infection and gut microbiota changes

#### 2.3.1 Gut microbiota changes and colorectal cancer

The term microbiome encompasses the community of microorganisms in a specific habitat, their structural elements, metabolites, and environmental conditions (Berg et al., 2020). The gut microbiota harbors a large population of microorganisms in close contact with the intestinal and colonic epithelium. It regulates important functions, such as food digestion, metabolism, immunomodulation, and colonization resistance against pathogens. As the intriguing host-microbiota relationship is crucial for maintaining homeostasis at the intestinal barrier sites, changes in the bacterial community structure and function have been proposed to be involved in CRC development, progression, and response to treatment (Hou et al., 2022). Some bacterial species are directly associated with an increased or reduced risk for colon tumorigenesis, but for many, exact mechanisms are not yet elucidated. Potential bacteria-host interactions in CRC could involve metabolic alterations, direct attachment, and invasion of bacteria into the tissue, as well as interactions with immune cells and, thus, triggering chronic inflammation (Ternes et al., 2020). *Fusobacterium nucleatum*, enterotoxigenic *Bacteroides fragilis*, and polyketide synthase (pks)+ *Escherichia coli*, for example, have been found in colonic tissues and in high abundance in the fecal microbiome of CRC patients. These species can promote malignant changes in the colonic epithelium by inducing inflammation or DNA damage (Boleij et al., 2015; Pleguezuelos-Manzano et al., 2020; Xu et al., 2021).

On the other hand, short-chain fatty acid (SCFA: butyrate, propionate, acetate) producing bacteria exert beneficial effects on the host by contributing to a balanced immune response and increasing mucus production in the gut (Song et al., 2020). Intestinal homeostasis requires a delicate balance between the microbiome, immune cells, and the epithelial compartment. Therefore, epithelial cells in the gut are covered by a mucus layer as a defense line, which separates bacteria from the host. The mucus layer consists of highly glycosylated mucus proteins secreted by goblet cells and forms a special niche and nutrient source for commensal microbes. Disturbance of the mucus layer or goblet cells and aberrant mucin production are linked to intestinal infections, inflammation, and, consequently, to CRC (Coleman and Haller, 2021; Gustafsson and Johansson, 2022).

#### 2.3.2 *H. pylori* infection and changes in the gastric microbiota

Microbiota changes associated with *H. pylori* infection have been extensively studied in the stomach, the site of infection. In general, different stimuli from the host, environment, lifestyle, or infections, such as *H. pylori*, can impact the gastric microbiota and drive the microbial composition towards a dysbiotic state. This dysbiosis then actively contributes to gastritis, gastric ulcers, and finally, gastric cancer. In addition, along the progression from a normal to malignant gastric epithelium, the microbial composition changes and is specific for the different states (Wang et al., 2023). Ferreira et al. have shown that patients with gastric cancer display a very different microbial community with genotoxic potential, which is even distinct from gastritis patients (Ferreira et al., 2018). At the same time, it was shown in actively infected humans that *H. pylori* is the dominating bacterium in the stomach and highly influences the overall composition (Schulz et al., 2018). Moreover, the gastric microbiota of *H. pylori*-infected patients had a significantly lower alpha-diversity than *H. pylori*-negative patients (Ndegwa et al., 2020). Using the insulin-gastrin (INS-GAS) transgenic mouse model, it was demonstrated that *H. pylori*-infected germ-free INS-GAS mice displayed fewer gastric lesions than *H. pylori*-infected mice colonized with a commensal microbiota, highlighting the interplay between *H. pylori*, other gastric microbes, and gastric cancer development (Lofgren et al., 2011; Kvin et al., 2014). These findings provide evidence that *H. pylori* infection affects the gastric microbial composition, which subsequently promotes gastric cancer risk. Finally, as a proof of concept, gastric microbiota from patients with intestinal metaplasia or gastric cancer transplanted into germ-free mice could reproduce major histopathological features of premalignant changes in the stomach of those mice, supporting evidence that *H. pylori* shapes a pro-carcinogenic gastric bacterial consortium (Kwon et al., 2022).

#### 2.3.3 *H. pylori* infection and changes in the intestinal microbiota

Although *H. pylori* colonizes the gastric mucus layer exclusively and strongly affects the gastric microbial composition, the infection is also associated with distant microbial changes in the small and large intestines (Schulz et al., 2018; Iino and Shimoyama, 2021). It is hypothesized that *H. pylori*-mediated shaping of the intestinal microbiota contributes to the increased CRC risk of infected hosts (Ralser et al., 2023). Several studies investigated the *H. pylori*-associated intestinal microbiota in both rodent models and humans.

Heimesaat et al. investigated microbiota changes along the entire GI tract of Mongolian gerbils after 14 months of *H. pylori* infection. The authors found distinct shifts of the microbiota composition in the large intestine alongside a pro-inflammatory response with increased CD3^+^ T-cell infiltration in the colonic tissue. The mucus-degrading species *Akkermansia*, known to contribute to reduced intestinal barrier function, was detected in the caecum and colon of *H. pylori*-infected mice. Higher abundances of *E. coli*, *Enterococcus* spp., and *Bacteroides/Prevotella* spp. were also detected in *H. pylori*-infected compared to non-infected control mice. Hypochlorhydria and hypergastrinemia of the stomach following *H. pylori* colonization were suggested to be responsible for those changes observed, allowing acid-sensitive bacteria to pass through the stomach and colonize the distant GI tract. Notably, the microbiome shifts depended on the presence of *H. pylori* CagA, which is in line with the CagA-dependent inflammation of the gastric and colonic mucosa. A chronic inflammatory state with T-cell recruitment, release of cytokines and chemokines, and epithelial barrier impairment might further contribute to a dysbalanced intestinal microbiome. However, the underlying mechanisms are not fully understood (Heimesaat et al., 2014). A similar shaping of the intestinal microbiota could be observed in wild-type mice infected for six months with the *H. pylori* PMSS1 strain. The microbial composition of fecal, cecal, and ileal samples of *H. pylori*-infected mice differed significantly from those of uninfected mice three months after infection, and these changes manifested over time. Members of the families *Turicibacteraceae*, *Erysipelotrichaceae*, and *Desulfobirionaceae* were identified to be altered in the large intestine upon *H. pylori* infection. At the same time, the infection upregulated expression of numerous genes involved in immune function in the stomach and lung tissues, highlighting immune crosstalk between *H. pylori* and distant organs (Kienesberger et al., 2016). The idea of strong immune-microbiome crosstalk across different organs in *H. pylori* infection was also supported by Fox et al., who found that wild-type mice from two different vendors with distinct gut microbial compositions showed different pathological, immunological, and gut microbial responses to *H. pylori* infection (Ge et al., 2018). Together, *H. pylori*-induced dysbiosis of the intestinal microbiota, related pro-inflammatory immune responses, and intestinal epithelial barrier dysfunction could be a possible explanation for the increased *H. pylori-*associated CRC risk. Also, *H. pylori* infection greatly influenced the intestinal microbiota of wild-type and *Apc*-mutant mice, and this *H. pylori*-shaped microbiota had a dominant effect on intestinal and colonic carcinogenesis. Compared to non-infected control mice, an expansion of *Akkermansia* spp. and *Ruminococcus* spp. was identified, both described as mucus-degrading taxa. At the same time, a significant loss of mucus-producing goblet cells was found, pointing towards impairment of intestinal barrier function and homeostasis. This can further promote inflammation and contribute to tumor development. *H. pylori*-infected germ-free *Apc*-mutant mice, however, did not only show a mild inflammatory phenotype but also a reduction of tumor numbers compared to SPF mice. Lastly, a stool transfer experiment from *H. pylori*-infected wild-type and tumor-prone mice into germ-free recipients alone was able to induce tumor development and inflammation in the small intestine and colon, proving the crucial role of an *H. pylori*-shaped intestinal bacterial community in CRC (Ralser et al., 2023).

In human patients, fewer studies investigated the influence of *H. pylori* infection on the gut microbiome compared to the gastric microbiome. In a German study, the fecal microbial composition of 212 *H. pylori*-infected patients and an equal number of negatively matched controls was analyzed using 16S rRNA sequencing. *H. pylori*-infected individuals had a significantly higher alpha-diversity than controls, which is usually an indicator of a healthy gut microbiome (Frost et al., 2019). An unchanged, or even higher, alpha-diversity of the intestinal microbiome in *H. pylori*-positive patients was also confirmed by several other studies, suggesting that *H. pylori* infection does not exert detrimental effects on the host by a general decrease of bacterial richness (Chen et al., 2018; Gao et al., 2018; Dash et al., 2019; He et al., 2019). However, regarding the overall bacterial composition, *H. pylori* positivity was associated with significant shifts. Thirteen taxa were significantly differentially abundant in *H. pylori*-positive cases compared to negative controls. Amongst them, *Prevotellaceae* abundance was enriched in *H. pylori*-infected patients, a taxa described as pro-carcinogenic (Yang et al., 2019a), and the facultative pathogen *Haemophilus*. In contrast, levels of *Pseudofavonifractor*, *Alistipes*, and *Fusicatenibacter*, all beneficial SCFA producers, were decreased compared to controls. In summary, gut microbial alterations associated with *H. pylori* infection may cause severe consequences for the host (Frost et al., 2019). In line with this, Dash et al. also demonstrated a differentially shaped *H. pylori*-associated intestinal microbiome with a potentially harmful capacity. Abundances of *Succinivibrio*, *Coriobacteriaceae*, *Enterococcaceae bacterium RF39*, and *Rikenellaceae* were increased in *H. pylori*-positive patients, and the authors linked those taxa with increased risk for intestinal barrier disruption and CRC development (Dash et al., 2019). Further, in a small cohort of patients, the fecal microbial composition of *H. pylori*-positive individuals was analysed in comparison with negative and eradicated patients. *H. pylori*-positive patients showed an increase in CRC-associated taxa, such as *Prevotellaceae* and *Peptostreptococcales*, further pointing towards a pro-carcinogenic *H. pylori*-shaped intestinal microbiota (Ralser et al., 2023). Gao et al. reported an association between the progression of *H. pylori*-induced gastric lesions and the fecal microbiome, especially for the decreased abundance of *Bacteroidetes* and increased abundances of *Firmicutes* and *Proteobacteria*. Interestingly, there was no remarkable difference in the fecal microbiota between current infected and eradicated patients (Gao et al., 2018). In addition, in the fecal microbiome of children with *H. pylori*-induced gastritis, higher abundances of *Betaproteobacteria*, *Lactobacillales*, and *Streptococcus*, and lower abundances of *Alphaproteobacteria* and *Megasphaera* compared to children with *H. pylori-*negative gastritis and healthy controls were identified. Most of the significant taxa belonged to gram-negative bacteria producing LPS, which might contribute to low-grade intestinal inflammation. Functional analysis further revealed that these different bacteria could also play a role in IBD and CRC (Yang et al., 2019b). Lactobacillus species, especially *Lactobacillus salivarius*, were highly increased in stool samples from *H. pylori*-infected subjects with severe atrophic gastritis compared to patients with mild or no gastritis or non-infected subjects (Iino et al., 2018). The same observation was made by Bühling et al., who found higher levels of *Lactobacillus acidophilus* in stool samples from *H. pylori*-positive individuals, which might be due to the influence of *H. pylori* on gastric acid secretion (Bühling et al., 2001). In addition, according to some other studies, gut microbiome alterations induced by *H. pylori* infection are positively linked to metabolic disorders, such as adiposity, type 2 diabetes, and hormone modulation (Martin-Nuñez et al., 2021). Remarkably, *H. pylori* eradication alleviated carbohydrate metabolism and normalized hormone secretion correlating with intestinal microbiome changes, which provides a proof-of-concept for *H. pylori*-mediated microbiome and metabolome modulation (Cornejo-Pareja et al., 2019). As metabolic disorders, in turn, are known to increase the risk for CRC, this might be a possible link between *H. pylori* infection and increased CRC incidence (Jin et al., 2022). Indeed, a study from 2010 supported a positive correlation between concomitant *H. pylori* infection and metabolic syndrome and the risk for colorectal adenomas (Lin et al., 2010). In summary, there is strong proof that *H. pylori* infection promotes changes in intestinal microbiota compositions, which can further contribute to CRC (**Figure 3**). Nevertheless, a molecular understanding of how the infection modulates bacterial compositions in distant organs is urgently needed.

## 3 Effects of antibiotic eradication of *H. pylori* on CRC risk

### 3.1 Epidemiologic studies

In 2020, a global consensus meeting was held in Taipei to discuss current evidence and therapeutic guidelines for *H. pylori* screening and eradication to prevent gastric cancer. The consensus report suggested offering all *H. pylori*-infected individuals eradication treatment. Different antibiotic regimens are available and should be chosen according to antibiotic resistance profiles, efficacy, adverse effects, and costs in the specific region (Liou et al., 2020). In general, standard treatment regimens comprise standard triple therapy (proton-pump inhibitor (PPI), most often omeprazole, clarithromycin, and amoxicillin), non-bismuth quadruple therapy (PPI, clarithromycin, amoxicillin, and metronidazole), or bismuth-containing quadruple therapy (PPI, bismuth, tetracycline, and metronidazole). In case of failure, fluoroquinolone-containing therapy (PPI, levofloxacin, amoxicillin, and, in case of quadruple therapy, bismuth) or PPI-amoxicillin high-dose dual therapy (PPI twice daily and amoxicillin) should be used (Malfertheiner et al., 2022). *H. pylori* eradication is an effective measure to reduce the risk of gastric cancer. A prospective, randomized controlled trial in a high-risk area in southern China found that *H. pylori* eradication treatment is associated with long-term protection against gastric cancer development (Yan et al., 2022). Furthermore, a systematic review and meta-analysis of twenty-four studies concluded that *H. pylori* eradication effectively reduces the risk for gastric cancer. In addition, the protective effect of eradication increased with increasing baseline gastric cancer incidences, arguing against a “point-of-no-return” in *H. pylori*-driven gastric carcinogenesis (Lee et al., 2016). Such “point-of-no-return” describes the fact that in some studies, *H. pylori*-positive patients presenting already with gastric intestinal metaplasia could not benefit from eradication, compared to patients with gastritis (Chen et al., 2016). The stage of intestinal metaplasia was therefore classified as “point-of-no-return”, suggesting imprinted immune and microbiome signatures in the stomach upon *H. pylori* infection. As some other studies drew different conclusions, a “point-of-no-return” in *H. pylori*-driven diseases is under debate.

Based on these studies, it might be speculated that *H. pylori*-positive patients could similarly benefit from antibiotic eradication regarding CRC development. In a retrospective study cohort of 615 adults in Taiwan, persistent *H. pylori*-infected patients had higher colorectal adenoma incidences than subjects without infection or those who underwent eradication therapy (60.11% vs. 20-24%). Therefore, *H. pylori* infection was associated with a significantly increased risk of colorectal adenoma compared to the eradication group (HR = 3.04), suggesting that antibiotic therapy may prevent CRC (Hu et al., 2019). Recently, Guo et al. conducted a large retrospective cohort study with almost 97.000 patients who had received a clarithromycin-containing triple therapy between 2003-2015 in Hong Kong. The authors compared the observed incidences of CRC in the eradication group with the expected incidences in the age- and sex-matched general population. While in the first five years after eradication, higher CRC incidences were observed (standardized incidence ratio (SIR) = 1.47), in the long-term follow-up after 11 years, eradicated study participants showed lower CRC incidences compared to the general population (SIR = 0.85), especially for rectal cancer (SIR = 0.90) (Guo et al., 2023). In conclusion, *H. pylori* eradication may confer protective effects and reduce the risk for CRC development in the long-term, at least as was shown in Asian populations. In this context, a new population-based cohort study, called “Nordic *Helicobacter pylori* Eradication Project”, with more than 670.000 patients in up to twenty-three years follow-up period will help to determine the impact of antibiotic therapy on gastrointestinal cancer risk in Scandinavian countries (Pettersson et al., 2023).

**Figure 4:**
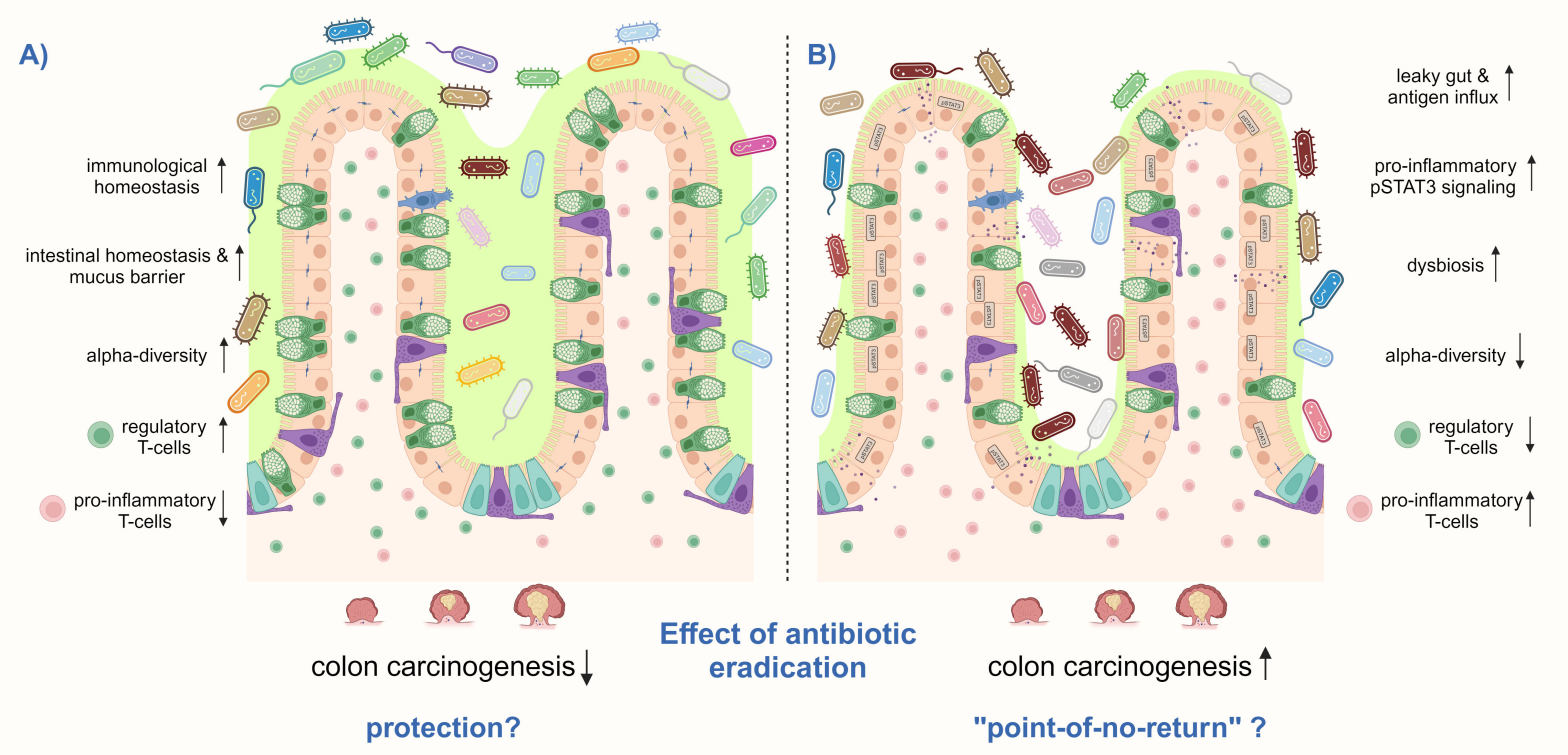
*H. pylori* eradication treatment can have different effects on the risk for CRC. **(A)** *H. pylori* eradication may prevent CRC by increasing and decrease the detrimental effects of the infection, particularly by reducing pro-inflammatory CD3^+^ T-cells and pSTAT3 signaling and increasing regulatory T-cells, thereby inducing immunological homeostasis in the small intestine and colon. In addition, the number of mucus-producing goblet cells may be restored, as well as the intestinal epithelial barrier function. Antibiotic treatment might only induce short-term intestinal dysbiosis, which finally recovers to a healthy composition with high microbial diversity. **(B)** However, there may also be a “point-of-no-return” in infected hosts, in which *H. pylori*-induced changes in the intestinal and colonic immune response and microbiome get imprinted and remain after antibiotic eradication. High levels of pro-inflammatory T-cells together with a low number of regulatory Treg cells may remain, as well as persistent activation of pSTAT3 signaling. Furthermore, antibiotic therapy leads to microbial dysbiosis and a reduction of alpha-diversity. These changes contribute to a leaky gut with antigen influx leading to a vicious pro-inflammatory cycle. Thus, colon carcinogenesis can not anymore be prevented by *H. pylori* eradication. Shown is a schematic representation of the intestinal epithelium.

A protective effect of *H. pylori* eradication on CRC would imply that *H. pylori*-induced changes in intestinal immune and microbiome signatures are reversible. Studies that address the intestinal immune response after *H. pylori* eradication therapy are lacking. In the up-to-date only experimental study available, Ralser et al. showed in *Apc*-mutant and wild-type mice that antibiotic eradication with a triple therapy regimen consisting of clarithromycin, metronidazole, and omeprazole after short-term *H. pylori*-infection, reduces tumor formation in the small intestine and colon to the level of non-infected controls, and prevents the *H. pylori*-induced phenotype of intestinal inflammation and barrier impairment. The analysis of colon tissue samples of 154 patients showed a declined phenotype in immune and epithelial signatures in *H. pylori*-eradicated patients. Moreover, eradicated patients showed a similar bacterial composition in their stool as non-infected patients, whereas the microbiota from actively infected patients clustered more distantly. This study further supports eradication therapy as a CRC prevention measure (**Figure 4**) (Ralser et al., 2023). However, Velin et al. administered antibiotics to *H. pylori*-infected mice one month before they were engrafted with B16-PVA melanoma cells and vaccinated to explore whether eradication improves the efficacy of anti-cancer vaccination. Eradication was found not to substantially revert the *H. pylori*-induced hyporesponsiveness to cancer immunity in mice (Oster et al., 2022). A similar imprinting of *H. pylori*-induced T-cell responses was observed when neonatal mice were either directly infected with *H. pylori* or treated with *H. pylori* immunomodulatory components and subsequently challenged in an allergy model (Kyburz and Müller, 2017). Although these findings were obtained in other experimental models than CRC, they suggest that *H. pylori*-imprinted changes in the immune system might not be reversible upon eradication, which significantly limits therapy options for the host (**Figure 3**). Therefore, if *H. pylori*-induced immune and microbiome changes in the small and large intestines after long-term infection are also imprinted or reversible upon antibiotic eradication must be further investigated to decipher optimal treatment options.

### 3.2 Effects of *H. pylori* eradication on gut microbiota

The scientific community’s attention to the effects of antibiotics on the gut microbiome has increased considerably. In general, antibiotics are more and more seen as potentially harmful because of their detrimental impact on the intestinal microbiota and related disorders, such as IBD (Ianiro et al., 2016) (Fishbein et al., 2023). High levels of antibiotic usage were also associated with an increased risk for colorectal neoplasia (Aneke-Nash et al., 2021). In addition, resistance rates of *H. pylori* to antibiotics have dramatically increased worldwide, but antibiotics are currently the only option to prevent *H. pylori*-related malignancies (Malfertheiner et al., 2022). However, one study provided evidence that the gut microbiota of young, healthy individuals is resilient towards a short-term broad-spectrum antibiotic therapy and could recover one and a half months after antibiotic treatment (Palleja et al., 2018). Ng et al. reported a remarkably high resilience and recovery capacity of the human microbiome after antibiotic treatment, where only short-term transient changes of some taxa were detected (Ng et al., 2019). Hence, elucidating alterations of the intestinal microbiota following antibiotic eradication is crucial to decipher and weight the risks and benefits of *H. pylori* eradication therapy on the host.

Several studies analyzing the intestinal microbiota after *H. pylori* eradication reported only short-term changes in alpha- or beta-diversity. After bismuth quadruple therapy, which is recommended first-line instead of triple therapy in high areas of clarithromycin resistance, temporary changes in the gut microbiota have been reported in adult patients with *H. pylori*-related gastritis. Antibiotics reduced the alpha-diversity and relative abundances of *Bacteroidetes* and *Actinobacteria* but increased that of *Proteobacteria*. However, already eight weeks post antibiotics, bacterial richness was restored to the levels at baseline, and at week 48, relative abundances of all phyla did not differ significantly from those before therapy. Here, patients reporting adverse effects during treatment also had higher abundances of *Proteobacteria* (Hsu et al., 2018). Another study, including sixty-three children from China, investigated short- and long-term changes in the intestinal microbiota after different *H. pylori* eradication regimens. Although short-term dysbiosis with reduced richness was observable after all antibiotic therapies (standard triple therapy including omeprazole, clarithromycin, and amoxicillin for fourteen days; sequential therapy including omeprazole plus amoxicillin for the first week, omeprazole plus clarithromycin and metronidazole for the second week; bismuth-based quadruple therapy including omeprazole and amoxicillin, metronidazole and bismuth for fourteen days, and concomitant therapy including omeprazole plus amoxicillin, clarithromycin, and metronidazole for fourteen days), one year after the antibiotic administration, richness, and composition recovered to baseline levels independently of the chosen antibiotics (Zhou et al., 2021). He et al. reported recovery of the gut microbiota already twenty-six weeks after bismuth quadruple therapy in ten young asymptomatic infected adults. No dysbiosis was seen in the long term. Instead, the beneficial taxa *Blautia* and *Lachnoclostridium* levels increased, the relative abundance of detrimental *Alistipes* decreased, and the overall composition approached that of healthy controls. As the intestinal microbiota after eradication differed from the *H. pylori*-associated microbiota before treatment, one could conclude that eradication treatment does not restore but rather abrogates *H. pylori*-induced microbial dysbiosis and thus could prevent *H. pylori*-associated diseases (He et al., 2019). Beneficial effects of antibiotic therapy in *H. pylori*-positive individuals were also observed in a prospective study from China analyzing stool samples from fifty-three *H. pylori* positive individuals by 16S rRNA sequencing. Successful *H. pylori* eradication enriched a probiotic *Bifidobacterium*-related taxa and did not substantially alter microbial diversity before and six months after receiving quadruple therapy (omeprazole, tetracycline, metronidazole and bismuth citrate for ten days) (Guo et al., 2020). Finally, in a multicentric, randomized trial including 1620 patients, only minimal adverse changes in fecal microbial diversity and composition were detected in the one-year follow-up after antibiotic treatment. *H. pylori* eradication also significantly improved metabolic parameters, for example, insulin resistance, triglycerides, and LDL levels (Liou et al., 2019).

However, some human studies also observed long-lasting and potentially harmful effects. A study with seventeen *H. pylori*-infected young adults aged eighteen to thirty years investigated the fecal microbiome before and after antibiotic eradication by 16S rRNA analysis. Whereas the overall richness and evenness of the intestinal microbiota did not differ much between before and after eradication, changes at the phylum level were found. In particular, the relative abundance of *Bacteroidetes* decreased, whereas *Firmicutes* increased until 12 months post-eradication, leading to a shift in the *Bacteroidetes/Firmicutes* ratio, which is associated with obesity. In addition, a significant increase in SCFA-producing bacteria was noticed, leading to an increased risk for metabolic disorders (Yap et al., 2016). A systemic review including 24 studies with three different follow-up periods reported inconsistent effects of *H. pylori* eradication on the intestinal microbiota. Alpha-diversity of fecal samples decreased in the short term, but no reliable conclusion could be drawn regarding the long-term observation of patients. While the authors found most phyla to recover to baseline levels at the long term, *Actinobacteria* levels, for example, decreased six months post-eradication compared to pre-eradication status. Together, these results suggest that antibiotic therapy for *H. pylori* eradication may affect microbial populations and richness beyond the stomach. These changes depend highly on race and eradication regimens (Ye et al., 2020). Long-term decreased relative abundances of *Actinobacteria* in the fecal microbiome of individuals receiving *H. pylori* triple therapy were similarly identified by Jakobsson et al. Still, alpha-diversity of the microbiota recovered to pre-treatment levels. However, in some individuals, a disrupted gut microbiome remained until up to four years post-treatment, indicating long-lasting consequences of *H. pylori* eradication on the intestinal microbiome (Jakobsson et al., 2010).

In conclusion, studies reported adverse effects of antibiotic therapy, with changes in the gut bacterial community lasting longer than a year. In many other studies, *H. pylori* eradication regimens appear safe, as no long-term consequences in alpha- or beta-diversity of the intestinal microbiota were identified (**Figure 4**). However, if we assume, based on current evidence, that *H. pylori* infection has a detrimental impact on the gut microbiota and that this dysbiosis may cause or contribute to inflammation and promote colorectal carcinogenesis, a “recovery” of the intestinal microbiota after *H. pylori* eradication treatment to the state before, is not favorable. Instead, the bacterial composition after eradication should convert to the composition of non-infected healthy subjects, as seen by (He et al., 2019). Recovery of the intestinal community to pre-eradication status would suggest that *H. pylori* infection of the stomach promotes changes in the intestinal microbiome, which get imprinted and persist even without an active infection. Hence, further studies are needed to address this question and its consequences.

## 4 Conclusion and perspective

Epidemiologic studies have demonstrated an increased risk for CRC development for *H. pylori-*infected subjects. With an OR of almost two, *H. pylori* infection appears to be a stronger risk factor for CRC development than other non-genetic predispositions, such as obesity (relative risk (RR) = 1.10), diabetes (RR = 1.42), smoking (RR = 1.08) and age (RR = 1.20) (Johnson et al., 2013; Ma et al., 2018). Assuming a prevalence of 40% and an OR of 1.8 for *H. pylori*-positive individuals to develop CRC, the population attributable risk (PAR) of CRC cases associated with *H. pylori* infection is 25-30%. Considering this PAR, nearly 110.000 CRC cases per year could be prevented in Europe by *H. pylori* prevention measures, for example through eradication therapy (Eileen et al., 2023). Hence, underlying mechanisms must be investigated to detect patients at high risk for CRC and subsequently intervene.

Modulation of the intestinal immune response through immunological cross-talk between the upper and lower GI tract, as well as shaping of the intestinal microbiota, emerge as the main mechanisms underlying *H. pylori*-induced CRC. Although *H. pylori* infection does not change overall microbial richness, the infection promotes microbial dysbiosis in the small and large intestines with an increase in pro-carcinogenic and mucus-degrading bacteria and a decrease of beneficial metabolite-producers. Considering the link between *H. pylori* and metabolic diseases and their impact on colon carcinogenesis, the identification of bacterial and corresponding metabolic signatures will be important to identify potential means of intervention.

Notably, the effects of antibiotic eradication therapy are still controversially discussed, and a possible protective effect of eradication therapy on the risk for *H. pylori*-driven CRC is relatively small. Based on the current scientific evidence, antibiotic therapy may be a potential intervention to prevent colon carcinogenesis in *H. pylori*-infected individuals. Nevertheless, more prospective studies in Western countries are urgently needed to confirm these findings and investigate the impacts of *H. pylori* eradication on CRC risk after long-term, chronic infection. A “point-of-no-return” of *H. pylori*-induced immune and microbiome signatures in the absence of active infection must also be considered. Especially regarding the intestinal microbiota and increasing *H. pylori* resistance rates, a careful evaluation of the risks and benefits of antibiotic treatment should be conducted.

The presence of bacterial virulence factors like CagA or VacA seems to play a significant role in *H. pylori*-driven intestinal carcinogenesis. *H. pylori* CagA-positive strains were associated with an increased risk for gastric cancer. Whether this pro-tumorigenic effect also accounts for CRC is still unclear, while potential underlying molecular mechanisms still must be elucidated.

In the future, more large-scale and prospective clinical studies are necessary to investigate the effect of active *H. pylori* infection, virulence factors, and antibiotic eradication on CRC risk. Deeper phenotyping of *H. pylori*-induced changes in intestinal immune responses, microbiome, and metabolome will undoubtedly help to unravel the exact mechanisms underlying gastric *H. pylori* infection and colonic carcinogenesis.

## Author contributions

VE: Writing – original draft. MG: Writing – review & editing. RM-L: Writing – review & editing.
